# Effect of Trinucleotide Repeats in the Huntington's Gene on Intelligence

**DOI:** 10.1016/j.ebiom.2018.03.031

**Published:** 2018-03-30

**Authors:** Jessica K. Lee, Amy Conrad, Eric Epping, Kathy Mathews, Vincent Magnotta, Jeffrey D. Dawson, Peg Nopoulos

**Affiliations:** a Department of Psychiatry, University of Iowa Carver College of Medicine, Iowa City, IA, United States; b Department of Pediatrics, University of Iowa Carver College of Medicine, Iowa City, IA, United States; c Department of Neurology, University of Iowa Carver College of Medicine, Iowa City, IA, United States; d Department of Radiology, University of Iowa Carver College of Medicine, Iowa City, IA, United States; e Department of Biostatistics, College of Public Health, University of Iowa, Iowa City, IA, United States

**Keywords:** Huntington's gene, Intelligence, CAG repeats, Evolution

## Abstract

**Background:**

Huntington's Disease (HD) is caused by an abnormality in the *HTT* gene. This gene includes trinucleotide repeats ranging from 10 to 35, and when expanded beyond 39, causes HD. We previously reported that CAG repeats in the normal range had a direct and beneficial effect on brain development with higher repeats being associated with higher cognitive function. The current study now expands this line of inquiry to evaluate the effects of CAG repeat throughout the entire spectrum of repeats from 15 to 58.

**Methods:**

We evaluated brain function in children ages 6–18 years old. DNA samples were processed to quantify the number of CAG repeats within *HTT*. Linear regression was used to determine if number of CAG repeats predicted measures of brain function.

**Findings:**

The number of repeats in *HTT*, had a non-linear effect on a measure of general intelligence with an inverted U shape pattern. Increasing repeat length was associated with higher GAI scores up until roughly 40–41 repeats. After this peak, increasing repeat length was associated with declining GAI scores.

**Interpretation:**

*HTT* may confer an advantage or a disadvantage depending upon the repeat length, playing a key role in the determination of intelligence, or causing a uniquely human brain disease.

## Highlights

• The *HTT* gene includes trinucleotide repeats ranging from 10 to 35 in the normal population.
• Huntington’s disease is caused when the trinucleotide repeats in the *HTT* gene expand beyond the normal range.
• The gene is vital for brain growth in which each repeat contributes to the development of brain function, measured as intelligence.
• Increasing repeats are beneficial to a certain point, then becomes detrimental to intelligence, forming an inverted U relationship.

A Faustian Bargain: Could the key to the evolution of the human brain be found in a dreadful disease? Huntington's Disease is a fatal progressive brain disease caused by a single gene. Results from the current study show that the same gene is important for brain development and intelligence. Therefore, this gene may have a simultaneous advantage and disadvantage: a role in the evolution of a superior human brain, yet the disadvantage of a uniquely human brain disease.

## 1 Introduction

The Huntingtin gene (*HTT*, OMIM 613004), is within a class of genes which contain a key region of simple sequence repeats (SSRs). The number of repeats typically ranges from around 10 to 35. However, when the number of repeats reaches 40 and above, the fatal neurodegenerative Huntington's Disease (HD) occurs. *HTT* has been shown to be critical for brain development (Cattaneo et al., 2005). Furthermore, SSRs have been hypothesized to play a vital role in evolution (Frenkel and Trifonov, 2012) by providing the variability needed to enhance changes of brain development (Hannan, 2010b). We recently reported the effects of *HTT* on brain structure and function in a large cohort of children with CAG repeats below disease threshold. We showed that the number of repeats in *HTT*, below disease threshold (15–35), confer advantageous changes in brain structure and general intelligence (IQ): the higher the number of repeats, the greater the change in brain structure, and the higher the IQ (Lee et al., 2017).

Although the current concept of the pathoetiology of HD is that of a neurodegenerative disease, mounting evidence suggests that abnormal development may play a vital role in the pathology (Nopoulos, 2016). This concept proposes that mutant *HTT* (*mHTT*) adversely affects brain development, creating circuits that are abnormal but remain functional in early life likely due to compensatory mechanisms (Arteaga-Bracho et al., 2016). If this concept is true, then evaluation of the effects of *mHTT* in the disease range may show evidence of abnormal brain development. It is well known that the number of CAG repeats above disease threshold has a dose-dependent effect with longer repeats manifesting in earlier onset of disease (Lee et al., 2013). In a manner similar to the effects below disease threshold, it is hypothesized that increasing lengths of CAG would have increasing effects of maldevelopment. Taken together, this suggests that when evaluating the effects of *HTT* along the entire spectrum – from below disease threshold through above disease threshold, a non-linear inverse U shaped curve might be expected where increasing lengths below disease threshold have an advantageous effect with a positive slope of change, yet increasing lengths above disease threshold have a disadvantage creating a negative slope of change in relation to increasing repeat lengths.

In a study designed to evaluate children (ages 6–18 years) at risk for HD (called the Kids-HD study), we extend our previous study of brain development in children with CAG repeats below disease threshold to include the entire spectrum of repeats (15–58, in our data), focusing on measures of cognition, motor skill and behavior. Our findings show that the effects of CAG repeat are advantageous in the range below disease threshold yet increasing repeat lengths are detrimental above disease threshold.

## 2 Methods

### 2.1 Participants

The sample was composed of children at risk for HD, and a healthy control sample. For the children at-risk cohort, adults who have been clinically diagnosed as having HD, or tested gene positive, were asked to enroll their children in the age range of 6–18 years. These families came from all over the United States. For the healthy control sample, children ages 6–18 were recruited from the local community by advertisements. Exclusion criteria for all subjects were the history of a major neurologic illness, brain surgery, or significant head trauma. All participants and their guardians signed informed consent prior to enrolling in the protocol, which was approved by the University of Iowa Institutional Review Board (IRB).

Testing for CAG repeat length was done using DNA from blood or saliva*.* Size of the CAG repeat region was determined with PCR analysis. PCR primers that exclude the adjacent polymorphic CCG tract were used to amplify the CAG region. A second set of primers that includes the CCG polymorphism is routinely used to assist in differentiating two alleles with an identical CAG repeat number. The CAG repeat length for each subject is determined by comparing the PCR products to sizing standards. Testing results were for research purposes only and were not released to study participants, their family, or members of the research team.

Table 1 displays the demographic and CAG repeat data. A previous analysis and publication of the effects of CAG repeat length in children below disease threshold utilized a total of 211 subjects (75 gene non-expanded and 136 healthy controls) (Lee et al., 2017). The current analysis expands this by adding 103 at-risk children and 2 healthy controls for a total of 316 children. The at-risk sample is divided into those who are Gene Expanded (GE, CAG repeat ≥40) and Gene Non-Expanded (GNE, CAG repeat ≤39). There were a total of 74 GE individuals and 104 GNE individuals.

**Table 1:** Demographics of sample.

	At risk		Healthy controls	Combined at risk and healthy controls
				Total
	Gene expanded (GE)	Gene non-expanded (GNE)	*N*	*N*
Number of individuals	74^b^	104	138	316
Additional (return) visits	42^b^	81	36	159
Total observations	116	185	174	475
Female/male	76/40	107/78	88/86	271/204

	***Mean (s.d.)***	***Mean (s.d.)***	***Mean (s.d*.)**	***Mean (s.d.)***
	**Range**	**Range**	**Range**	**Range**
Age (years)	13.0 (3.6)	13.0 (3.5)	12.4 (3.5)	12.8 (3.6)
	6.0–18.9	6.0–18.9	6.0–18.9	6.0–18.9
CAG repeat	44.5 (4.6)	20.5 (4.0)	20.3 (4.00)	26.7 (11.4)
	40–58	15–39	15–31	15–58
Parental SES^a^	2.63 (0.65)	2.66 (0.69)	2.27 (0.45)	2.51 (0.63)
	2–5	2–5	1–3	1–5

a Parental Socioeconomic Status (SES) based on a modified Hollingshead scale of 1–5 with the higher the number, the lower the status.

b Six GE subjects removed from analysis with CAP Inclusion criteria ≥0.68, 2 additional return visits removed with CAP Inclusion criteria ≥0.68/ exclusion >0.67.

#### 2.1.1 Refining the Sample to Exclude Disease Process

Studies of preHD adults have shown that as far back as more than 10 years prior to disease onset, changes in the brain are detected (Ross et al., 2014). One method to predict the time from assessment to onset of disease is the calculation of a CAP score (CAG-age product) which is a proxy variable for time to diagnosis. CAP is computed by multiplying age at study entry (Age_0_) by a scaling of the CAG repeat length (CAP = Age_0_ × (CAG − 33.66)/432.3326). A low CAP score of <0.67 represents individuals who are far from onset (roughly 12 years) while subjects in the medium group of >0.67 and < 0.85 are roughly 7.5 years from onset and those with a CAP score >0.85 are estimated to be less than 5 years to onset (Zhang et al., 2011). Importantly, cognitive changes can be seen many years prior to disease onset which is defined by motor abnormality. In a large study of nearly 600 preHD subjects, it was shown that the low CAP score group showed no significant cognitive change compared to gene negative controls in any measure assessed. However, both medium and high CAP score groups showed significant deficits of cognitive test scores compared to controls, demonstrating the effect of the disease process happening even prior to motor onset (Harrington et al., 2012). In the current study, the goal is to capture a measure of brain function that reflects brain development and not disease (especially the cognitive changes seen in the time prior to motor onset). In order to minimize the effects of the disease process, we excluded any participant with a CAP score of >0.67. This removed a total of 6 individuals with CAG repeat lengths ranging from 54 to 59, age at assessment ranging from 12.7 years to 18.5 years and CAP scores between 0.71 and 0.89. A higher CAP score could reflect either higher age or higher CAG repeat. For instance, in our data, a CAP score of 0.31 is represented both by a 7.7 year old with a repeat of 51 and an 18.3 year old with a repeat of 41. Therefore although there remained many subjects with repeat lengths >50, in order for them to have a CAP score <0.68, they would have had to be assessed at a relatively young age in order to be considered far from onset and not yet in the disease process. Indeed, the average age of GE subjects with CAG repeat greater or equal to 50 is 9.4 years (*s.d*. 1.9) compared to the average age of those with repeat lengths between 40 and 49, at 13.3 years (*s.d*. 3.5).

As our protocol is longitudinal, several subjects returned for repeat assessments: In the at-risk group, the GE group had 42 return visits for a total of 116 observations. After exclusion of observations with CAP scores >0.67 2 return visits were removed. The GNE group had 81 return visits for a total of 185 observations. In the control group, there were 36 returns for a total of 174 observations. The mean interval between return visits was 24.9 months (*s.d. *= 9.94). After removal of subjects with CAP score >0.67, total number of subjects = 310, total additional return visits = 157 for total number of observations = 467. The average age of GE and the GNE groups was 13.0 with a range of 6.0 to 18.9 years of age, and the average age of the controls (174 observations) was 12.4 with a range of 6.0–18.9 years of age. Parental Socioeconomic Status (SES) was determined by a modified Hollingshead Scale (1–5 where lower numbers mean higher SES), with a mean of 2.63 (*s.d.* = 0.65) for the GE sample, 2.66 (*s.d*) = 0.69 (for the GNE sample, and 2.27 (*s.d.* = 0.45) for the control sample.

### 2.2 Functional Domains

General Ability Index (GAI) was determined by Wechsler Intelligence Scale for Children-4th Edition (WISC-IV; for ages 6–16 years) and Wechsler Adult Intelligence Scale, 4th Edition (WAIS - IV; for ages 17–18 years).

The General Ability Index (GAI) is a composite of Verbal Comprehension Index (VCI) and Perceptual Reasoning Index (PRI) used as an estimate of global intelligence.

A battery of cognitive tests designed to assess specific areas of skill was administered. To minimize number of comparisons, skill domains were constructed by identifying a group of tests that measured specific functional skill within that cognitive domain. Raw scores were utilized and for consistency, all scores were first transformed into z scores based on the mean of the group. For scores in which a greater score represented worse performance, the sign of the metric was switched so that higher meant better. Finally, a composite average of the z-scores was calculated to determine the domain score, with a resulting domain score in which higher scores indicate better performance. If any test was missing, then the score for that domain was not calculated and therefore not used in the analysis. Tests for the Language domain included the Boston Naming Test (Kaplan et al., 1983) and the phonemic verbal fluency, and free sorting description within the Delis-Kaplan Executive Function System (Delis et al., 2001) (D-KEFS). Tests for the Visual-Perceptual domain included the Benton Judgment of Line Orientation Test (Benton et al., 1994) and the Bender Developmental Test of Visual-Motor Integration (Brannigan, 2003). The Executive Function domain was comprised of scores from the D-KEFS color word interference, verbal fluency switching, and sorting tasks; and omissions and commissions errors of the Continuous Performance Task (Conners, 2000). Tests that constituted the Memory domain included the Wechsler spatial span, the Children's Memory Scale (Cohen, 1997) and the Color Span test (Richman and Lindgren, 1988). A Motor domain was comprised of measures from the Physical and Neurologic Evaluation of Subtle Signs (PANESS) which assess motor function including gait, balance, motor persistence, coordination, overflow, dysrhythmia, and speed (Larson et al., 2007). A Behavior domain was calculated from total scores of the Pediatric Behavior Scale (PBS) (Lindgren and Koeppl, 1987), a parent rating scale, and the Behavior Rating Inventory of Executive Function (BRIEF), a parent rating scale specifically geared toward evaluation of executive function (Gioia et al., 2002).

### 2.3 Statistical Analysis

A typical longitudinal study analysis was not conducted meaning that subjects were not evaluated in how they changed from time 1 to time 2. Instead, the strategy was to use both baseline and repeat visits in order to create the largest data base of observations. Then, these observations were used to predict a variable of interest, controlling for the correlation that comes with repeat measures of the same person. This is a method that is considered gold standard in the assessment of childhood samples spanning large age ranges (Giedd et al., 2008).

**Fig. 1:**
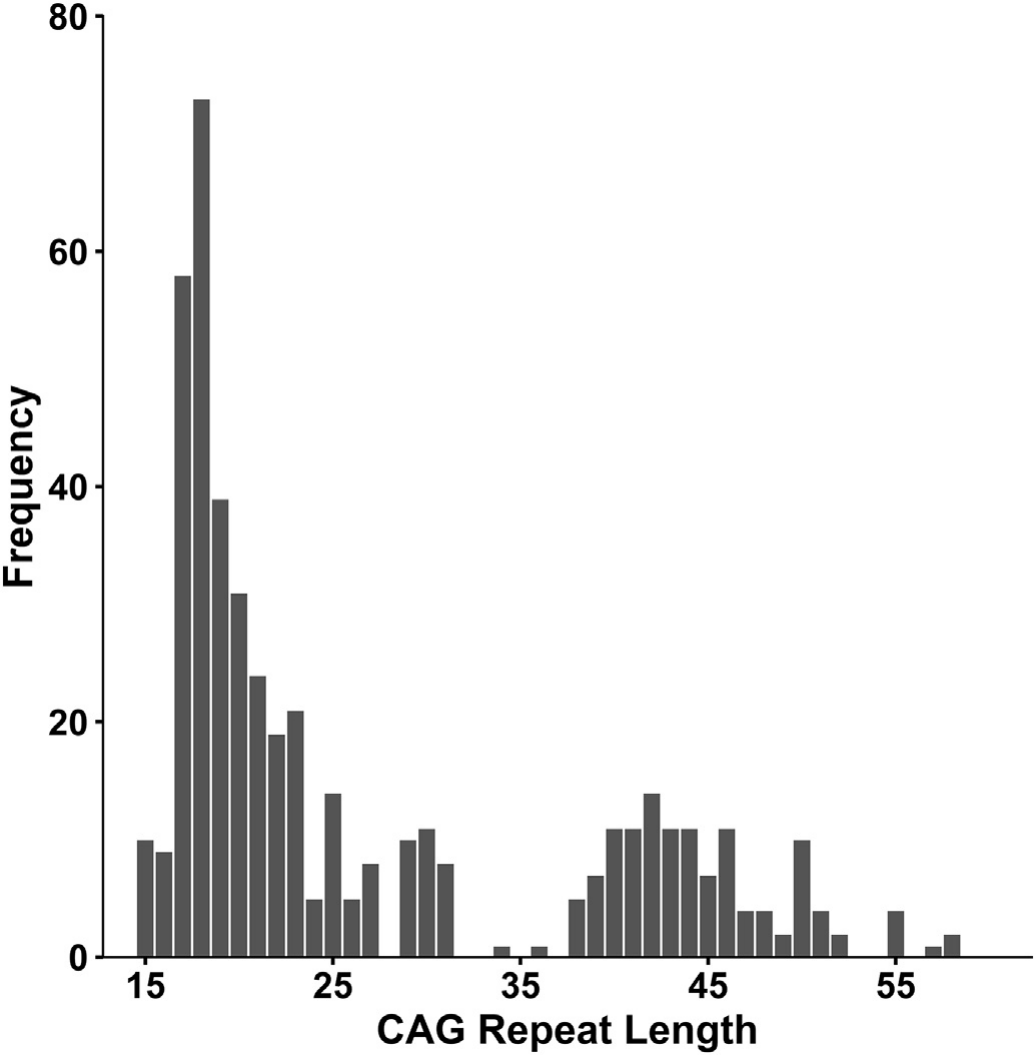
The frequency distribution of the CAG repeats in the longest allele.

All analyses were performed by using SAS/STAT procedures. The Mixed Procedure was used to run regression models while accommodating repeat visits to predict quantitative measures of general intelligence, and skill domain scores (dependent variables) based on CAG length (independent variable). Analysis was done first using the allele with the longest repeat and then repeated using the shorter allele. The distribution of CAG repeat number was not normal based on the frequency of allele lengths in the general population as well as those at risk for HD (see Fig. 1). Therefore, CAG length was log transformed for normalization, and this was used as the primary predictor of interest, both in linear and non-linear (quadratic) models. Age, sex, and parental socioeconomic status (SES) were controlled for due to their impact on brain function. The model considered all potential interactions as well as both linear and non-linear age relationships, which were dropped from the model if not significant. All domain scores (with the exception of behavior) were correlated with GAI, (Pearson's *r* controlling for age, sex, parental SES): Language *r* = 0.61 (*p* < .01), Visual-Perceptual *r* = 0.53 (*p* < .01), Executive *r* = 0.41 (*p* < .01) Memory *r* = 0.44 (*p* < .01) and Motor *r* = 0.20 (*p* < .01). Therefore, the model of these measures additionally controlled for GAI in order to assess the effect of CAG repeat length above and beyond that contributed by general intellect (again, the exception was for behavior where GAI was not included as a covariate). A 2-tailed alpha level of 0.05 was used for significance tests.

For visualization purposes only, the data set was divided into bins of CAG repeat length, and any significant model was re-run with CAG bin as a group variable, so that means and standard errors (*s.e.*) could be plotted for each bin. The bin sizes were determined so that comparable number of samples were represented in each bin, across the CAG length spectrum. The observations below disease threshold comprised a large portion of the sample (359 compared to 109 above disease threshold). Therefore the first 2 bins contained those with CAG repeats 15–19 (*n* = 189), and repeats 20–39 (*n* = 170). Those above disease threshold were binned in the following manner: 40–41 repeats (*n* = 22), 42–44 repeats (*n* = 25), 45–48 repeats (*n* = 37), and 49–58 repeats (*n* = 25).

## 3 Results

**Fig. 2:**
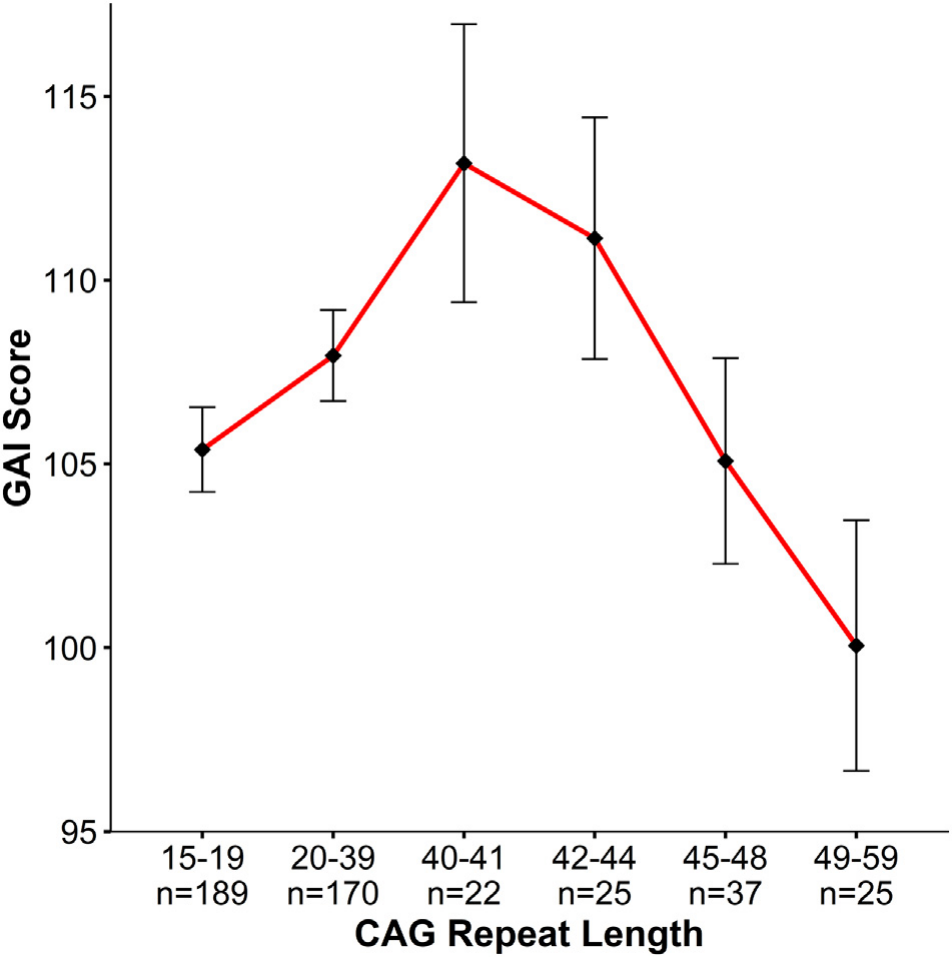
General abilities index (GAI). Graph above shows results of the non-linear model (β = −20.2, *p* = .006) where the x-axis is represented by groups of subjects binned by CAG repeat length of the longest allele, and the y-axis is the mean GAI (bars are standard error) for each group. To obtain mean GAI, ANCOVA was performed between groups, controlling for age, sex, and parental SES.

Table 2 displays the results of the model analysis with respect to effects of CAG, including all variables (and interactions) in the foot notes included in the final model. There was no linear effect of CAG repeat on GAI (β = 1.04, *p* = .61), however there was a strong non-linear effect which was negative (β = −20.2, *p* = .006), indicating an inverted U-shaped curve. Fig. 2 illustrates this relationship with increasing repeats associated with higher GAI scores up until the bin of 40–41 repeats. After this peak, the average GAI decreased for each subsequent group with longer average CAG repeats. Although this illustration suggests that the peak GAI score is in children who are within the disease producing range, it is important to caution against defining exactly where the peak effect is given the low numbers of subjects in the groups 36–39 (*n* = 13 observations) and 40–41 (*n* = 22 observations).

**Table 2:** Results of analysis of brain function measures predicted by CAG repeat length, long allele.

Kids-HD sample^b^	CAG repeat length			
	Slope from linear model		Quadratic term from non-linear model	
	β (*s.e*.)	*p*	β (*s.e.*)	*p*
WISC-IV and WAIS-IV^a^ (*n* = 467)				
General abilities index (GAI)^d^	1.04 (2.05)	0.612	−20.2 (7.3)	0.006
Domain Z scores^c^				
Language (*n* = 440)^e^	0.00 (0.06)	0.968	−0.35 (0.22)	0.102
Visual perceptual^f^ (*n* = 438)	0.22 (0.07)	0.004	0.13 (0.276)	0.631
Executive^g^ (*n* = 399)	−0.04 (0.06)	0.561	0.02 (0.24)	0.917
Memory (*n* = 424)^h^	−0.02 (0.07)	0.76	−0.20 (0.25)	0.420
Motor^i^ (*n* = 455)	0.00 (0.07)	0.991	−0.23 (0.28)	0.37
Behavior^j^ (*n* = 445)	−0.22 (0.13)	0.104	−0.21 (0.49)	0.675

a Wechsler Intelligence Scale for Children-4th Edition and Wechsler Adult Intelligence Scale, 4th Edition.

b Analysis controlled for age, sex and parental socioeconomic status (SES). In addition, for all domain scores except behavior, GAI was controlled for in order to account for effects of general intellect.

c Number of observations indicated in parentheses after accounting for missing data in calculation of domain score.

d Primary effect of parental social class.

e Primary effect of age and age ∗ age interaction, sex effect F > M.

f Primary effect of age and age ∗ age interaction, sex effect M > F.

g Primary effect of age, sex effect F > M.

h Primary effect of age and age ∗ age interaction.

i Primary effect of age and age ∗ age interaction, sex effect F > M.

j Primary effect of age and age ∗ age interaction, sex effect F > M.

Parental socioeconomic class had a primary effect on GAI and domain scores of language and behavior with all effects showing association between higher socioeconomic class and higher scores (i.e. better language performance and less problematic behavior). There was no effect of age on GAI (*F* = 0.05, *p* = .83), consistent with literature that reports developmental stability of intelligence measures (Burgaleta et al., 2014; Deary et al., 2000). However, there were primary effects of age on all domain measures with higher scores being associated with increasing age. Also, all domain scores, except for behavior had a non-linear age effect in which there was a steep upward slope in scores with age until approximately 16 years of age, where the slope became gradual, indicating a slowing of age effects past 16 years. Females had higher scores than males in domains of language, executive, motor, and behavior while males had higher scores in the visual-perceptual domain. There were no interactions between sex, CAG repeat and GAI or any domain score.

**Fig. 3:**
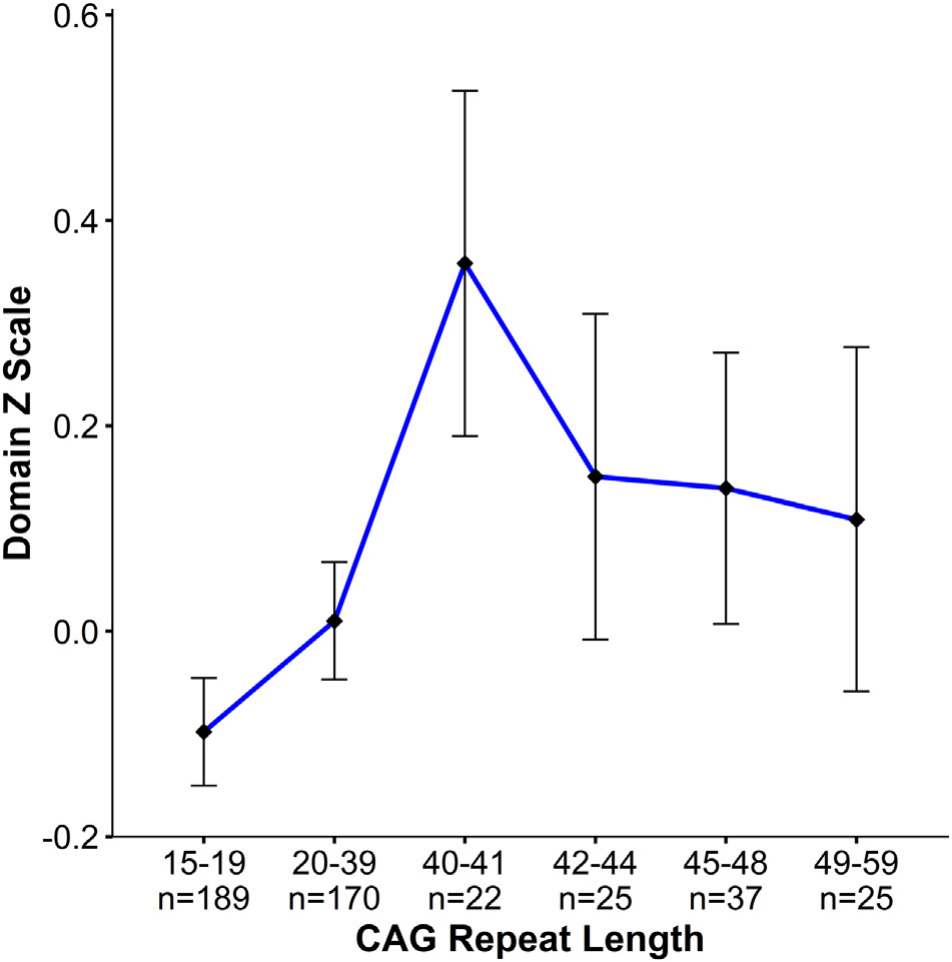
Visual perceptual domain score. Graph above shows results of the linear model for the visual-perceptual domain z score (β = +0.22, *p* = .004) where the x-axis is represented by groups of subjects binned by CAG repeat length of the longest allele, and the y-axis is the mean domain z score (bars are standard error) for each group. To obtain mean visual-perceptual domain score, ANCOVA was performed between groups, controlling for age, age ∗ age, sex, GAI and parental SES.

In regard to the domain scores, also shown in Table 2, the only significant relationship between CAG repeat length and functional measure was with the linear model for the visual-perceptual domain score which was positive (β = +0.22, *p* = .004) indicating that higher repeats were related to higher scores. This relationship is shown in Fig. 3 in which the domain scores increase from the lowest at the15–19 repeat group to a peak at the 40–41 repeat group. After that, there is a modest decline and plateau across the remaining end of the repeat spectrum. Although the model is linear and the non-linear quadratic coefficient for this variable was not significant, the pattern suggests a beneficial effect on visual-perceptual skills of repeats up to 40–41 repeats.

All models repeated using the shorter allele were non-significant indicating it is the longer allele that acts in a fully dominant fashion.

A post-hoc analysis was conducted to quantify the percentage of variance in GAI scores accounted for by CAG repeat. The sample was split into two overlapping sections: to reflect both the advantageous effects (CAG repeats 15–41) and the deleterious effects (CAG repeats 42 and above). These samples were then run using the same linear model as before. Estimates of the variance explained by CAG repeat length were calculated by the R^2^ multiplied by 100, expressed as a percentage. The R^2^ was calculated to be the proportion of variance accounted for by the regressors in the model, calculated using the residual variance of the full model (*V*_*full*_) and the residual variance of the model without the regressor of interest (*V*_*null*_) (Selya et al., 2012): $R^{2}=\frac{V_{\mathrm{null}}-V_{\mathrm{full}}}{V_{\mathrm{null}}}$. Table 3 shows the results of this analysis. The advantageous effects of CAG on GAI in the range of 15–41 repeats accounts for 1.5% of the variance while the deleterious effects of CAG on GAI in the range of ≥ 42 is an even stronger effect, accounting for 12.9% of the variance.

**Table 3:** Results of the analysis calculating the percentage of variance in GAI score accounte.

	β (*s.e*.)	*p*	% Variance explained by CAG repeat
Advantageous effect on GAI (CAG repeat 15–41)	+6.78 (3.41)	0.051	1.5%
Deleterious effect on GAI (CAG repeat ≥42)	−54.23 (16.45)	0.002	12.9%

## 4 Discussion

Our previous study found that increasing numbers of triplet repeats in *HTT*, within the normal range, confer advantageous changes in the function of the developing human brain, with more repeats being directly related to superior cognitive function. The current analysis expands these findings by showing that within the disease causing range, the relationship is inverse – the greater the number of repeats, the worse the cognitive function. Therefore, the effects of CAG repeats in *HTT* may have simultaneous advantage and disadvantage, forming a non-linear inverted U-shape relationship with advantageous changes occurring below disease threshold and once above disease threshold, increasing CAG repeats results in aberrant development manifesting in poorer cognitive function. Where exactly the zenith of the curve is in regard to CAG repeats is not definitely addressed by this study as the lowest number of observations were in the 25–39 repeat range and larger samples could provide a more granular relationship to repeats within that range.

The effect of CAG repeat length was limited to the allele with the longest repeat as analysis evaluating the effect of the shorter allele was completely non-significant. This is consistent with other reports that have shown the allele with the longest CAG repeat to be fully dominant in both normal function below disease threshold as well as above disease threshold (Lee et al., 2012; Seong et al., 2005).

In regard to potential advantageous effects of *HTT* in the lower range of the spectrum, there has been speculation that triplet repeat genes may have been positively selected for in the evolution of the human brain. Work by Cattaneo has shown that *HTT* is highly conserved where greater number of repeats are found in more evolved species, with humans having the highest number (Tartari et al., 2008). This suggests that the high number of repeats is uniquely human and that the selective pressure is geared toward repeat polymorphism.

The notion that intelligence has a strong genetic influence has been known for over 100 years (Plomin and Deary, 2015), and general intelligence (IQ) is considered to be one of the most heritable behavioral traits in humans (Plomin et al., 2008). In contrast to the genetic studies that have identified genes having substantial effects on anthropometric measures such as height and weight, no studies have identified and replicated any gene, or gene variant, that significantly relates to intellect (Chabris et al., 2012). A recent Genome-wide association Study (GWAS) meta-analysis analyzing 35,298 individuals, found two novel loci that were associated with general cognitive function. Yet, the effect sizes are small with findings accounting for roughly 0.1% of the variance (Trampush et al., 2017). Importantly, GWAS studies identify genetic variation, but they do not quantify simple repeat sequences such as CAG repeats in *HTT*. Thus, the current study cannot be directly compared to that of a GWAS. However, when utilizing the same metric of effect (% of variance in intelligence accounted for by a genetic variant) the effect of CAG repeat on intelligence is far greater than the effect of any other SNP reported in the GWAS study. Repeat length between 15 and 41 CAG explained 1.5% of the variance in general intellect in the lower range of repeats (15–41). Although this effect remains small, it is 15 times as large as the effect recently reported in the GWAS meta-analysis. Moreover, the effect of *HTT* in the higher repeat range of ≥42 showed an even more robust relationship, accounting for 12.9% of the variance in general intellect.

Although there have been great strides in the field of genetics, the lack of studies that evaluate the effects of tandem repeat polymorphisms reveals an important area for further research. Some have suggested that the “missing heritability” identified after GWAS studies have failed to detect key sources of genetic variation can be found in the study of simple sequence repeats (Hannan, 2010a). Tandem repeat polymorphisms have been shown to be important in brain development in other species and these dynamic mutations could act as genetic modulators, in particular in the context of human brain evolution (Nithianantharajah and Hannan, 2007). In regard specifically to *HTT*, variation in repeats have been recently been shown to be associated with the lifetime risk for depression, also in a non-linear fashion (Gardiner et al., 2017). In addition, our group has previously reported that CAG repeat below disease threshold is associated with assortative mating (Nopoulos et al., 2011). Assortative mating on intelligence is a well-known phenomenon (Vinkhuyzen et al., 2012), and the findings here provide an additional level of support to the notion that CAG repeats may be related to intelligence, which in turn is related to assortative mating. The current study prompts future studies to examine polymorphic repeats and the associated genetic consequences on normal and pathologic brain development.

The children in this sample that are gene expanded are estimated at the minimum to be greater than 12 years from onset of the disease, with some children likely up to 30 years prior to onset (for example those younger than 10 with CAG repeats 40–44). Although the children with relatively low disease-causing range repeats (40–44) had beneficial effects of CAG repeat on their GAI score, those with repeats 45 and above had substantially lower GAI scores. In PreHD adults the substantial deficits in cognitive function detected in subjects designated to be in medium or higher CAP groups, are thought to be due to early phases of the degenerative process, while subjects with CAP scores <0.68 were found to be indistinguishable from controls and therefore had not yet entered the degenerative or active part of the disease (Harrington et al., 2012) This was the rationale for the current study in which any subject with a CAP score >0.68 was eliminated, minimizing the likelihood that any of the subjects were in the early stages of disease or symptom manifestation. Therefore the cognitive function measured for children with CAG repeats over the disease threshold is not likely due to active degenerative disease process, but rather reflective of an effect of *mHTT* on brain development. In an effort to support this further, the analysis was re-run, deleting any subject with a CAP score >0.49, dropping 17 observations, and minimizing further any possibility of children who were in an active degenerative phase of the disease contributing to the findings. The results of the analysis remained significant.

There is increasing support for the notion that abnormal brain development may play a vital role in the pathoetiology of HD (Nopoulos, 2016). The theory posits that the effect of *mHTT* is to impact development of a specific set of cells or circuit, which in the case of *mHTT* is likely the medium spiny neurons of the striatum. These cells, however, are initially compensated for in childhood rendering the circuit in a mutant steady state where no significant functional alterations are detectable despite the abnormal development. This developmentally aberrant circuit is therefore vulnerable such that environmental stressors as normal maturation and aging processes that may not typically lead to cell death would weaken the circuit causing dysfunction and eventually cell death and degeneration, later in life (Arteaga-Bracho et al., 2016; Mehler and Gokhan, 2000). Regardless of whether or not the effects of CAG repeat are advantageous or deleterious, it appears that *mHTT* is affecting the development of the brain in children destined to develop the disease, supporting the notion that this development sets the stage for later degenerative disease. In the case of children with repeats 40–44, early structure and function is quite good and there may be no need for compensatory mechanisms in childhood and onset of the disease occurs on average, at the age of 40 years. For those in the higher repeat range, development may be hampered, creating a more vulnerable circuit that, even with compensation, deteriorates with emergence of symptoms much earlier in life than those with shorter repeats.

The effects of *HTT* were strongest for general intelligence scores. After accounting for general intelligence, only specific advantages associated with CAG repeat length in visual-perceptual skills were detected. Similar to the effect on GAI, there were advantageous changes with increasing repeats up until the range of 40–41. Yet unlike GAI, there was no significant deleterious effect with increasing repeats above disease threshold. Early changes in tests of sensory and perceptual processing such as negative emotion recognition, smell perception, and performance on a timing task have been shown to be predictive of disease onset in preHD subjects (Harrington et al., 2012). However in the current study, the visual-perceptual tasks were measuring different sensory and perceptual processes, namely visual-motor integration (the Bender test) and pure visual-spatial perception (Judgement of Line). Interestingly, a recent report suggests that the Judgement-of-Line task, unlike other measures, was not found to be abnormal in patients until later in the disease process, after disease manifestation, suggesting it is a skill relatively spared (Corey-Bloom et al., 2016). The current findings in which there was no CAG repeat dosage effect on the Judgement-of-Line task performance above disease threshold is consistent with the previous findings of preserved visual-spatial perceptual skills in preHD.

In sum, the findings from the current study support the notion that *HTT* may confer an advantage or a disadvantage depending upon the repeat length, playing a key role in determining intelligence, or causing a uniquely human brain disease.

## Funding Source

This study was supported by the National Institutes of Neurologic Disorders and Stroke (NINDS) grant R01 NS055903, the National Center for Advancing Translational Sciences (NCATS) grant 1U54TR001356, the University of Iowa Clinical and Translational Science Award 1U54TR001013, and the CHDI Foundation grant 071108. The funders had no role in study design, data collection, data analysis, interpretation or writing of the report.

## Conflict of Interest

There are no identified conflicts for any author. All authors have submitted a Conflict of Interest Statement.

## Author Contribution

J.L. assessed subjects, maintained data base, performed statistical analysis and was primary author of manuscript; A.C. was involved in protocol development, assessment of subjects and statistical analysis. E.E. and K.M. were involved in protocol development and assessment of subjects; V.M. developed the MRI sequence protocol and image processing; J.D. was the lead statistician; P.N. provided oversight of protocol and scientific design. All authors discussed the results and commented on the manuscript.

## Ethics Committee Approval

This study was reviewed and approved by the Iowa Investigational Review Board (IRB).

## References

[bb0005] Arteaga-Bracho E.E., Gulinello M., Winchester M.L., Pichamoorthy N., Petronglo J.R., Zambrano A.D., Inocencio J., de Jesus C.D., Louie J.O., Gokhan S., Mehler M.F., Molero A.E. (2016). Postnatal and adult consequences of loss of huntingtin during development: implications for Huntington's disease. Neurobiol. Dis..

[bb0010] Benton A., Sivan A., Hamsher K., Varney N., Spreen O. (1994). Contributions to Neuropsychology Assessment: A Clinical Manual.

[bb0015] Brannigan (2003). Bender Visual-Motor Gestalt Test.

[bb0020] Burgaleta M., Johnson W., Waber D.P., Colom R., Karama S. (2014). Cognitive ability changes and dynamics of cortical thickness development in healthy children and adolescents. NeuroImage.

[bb0025] Cattaneo E., Zuccato C., Tartari M. (2005). Normal huntingtin function: an alternative approach to Huntington's disease. Nat. Rev. Neurosci..

[bb0030] Chabris C.F., Hebert B.M., Benjamin D.J., Beauchamp J., Cesarini D., van der Loos M., Johannesson M., Magnusson P.K., Lichtenstein P., Atwood C.S., Freese J., Hauser T.S., Hauser R.M., Christakis N., Laibson D. (2012). Most reported genetic associations with general intelligence are probably false positives. Psychol. Sci..

[bb0035] Cohen M.J. (1997). Children's Memory Scale. Administration Manual.

[bb0040] Conners C.K. (2000). Conners' Continuous Performance Test II: Computer Program for Windows Technical Guide and Software Manual.

[bb0045] Corey-Bloom J., Gluhm S., Herndon A., Haque A.S., Park S., Gilbert P.E. (2016). Benton judgment of line orientation (JoLO) test: a Brief and useful measure for assessing visuospatial abilities in manifest, but not Premanifest, Huntington's disease. J. Huntingtons. Dis..

[bb0050] Deary I.J., Whalley L.J., Lemmon H., Crawford J.R., Starr J.M. (2000). The stability of individual differences in mental ability from childhood to old age: follow-up of the 1932 Scottish Mental Survey. Intelligence.

[bb0055] Delis D., Kaplan E., Kramer J.H. (2001). Delis-Kaplan Executive Function System (D-KEFS).

[bb0060] Frenkel Z.M., Trifonov E.N. (2012). Origin and evolution of genes and genomes. Crucial role of triplet expansions. J. Biomol. Struct. Dyn..

[bb0065] Gardiner S.L., van Belzen M.J., Boogaard M.W., van Roon-Mom W.M.C., Rozing M.P., van Hemert A.M., Smit J.H., Beekman A.T.F., van Grootheest G., Schoevers R.A., Oude Voshaar R.C., Roos R.A.C., Comijs H.C., Penninx B., van der Mast R.C., Aziz N.A. (2017). Huntingtin gene repeat size variations affect risk of lifetime depression. Transl. Psychiatry.

[bb0070] Giedd J.N., Lenroot R.K., Shaw P., Lalonde F., Celano M., White S., Tossell J., Addington A., Gogtay N. (2008). Trajectories of anatomic brain development as a phenotype. Novartis Found. Symp..

[bb0075] Gioia G.A., Isquith P.K., Retzlaff P.D., Espy K.A. (2002). Confirmatory factor analysis of the Behavior Rating Inventory of Executive Function (BRIEF) in a clinical sample. Child Neuropsychol..

[bb0080] Hannan A.J. (2010). Tandem repeat polymorphisms: modulators of disease susceptibility and candidates for 'missing heritability. Trends Genet..

[bb0085] Hannan A.J. (2010). TRPing up the genome: tandem repeat polymorphisms as dynamic sources of genetic variability in health and disease. Discov. Med..

[bb0090] Harrington D.L., Smith M.M., Zhang Y., Carlozzi N.E., Paulsen J.S., Group, P.-H. I. O. T. H. S (2012). Cognitive domains that predict time to diagnosis in prodromal Huntington disease. J. Neurol. Neurosurg. Psychiatry.

[bb0095] Kaplan E., Goodglass H., Weintraub S. (1983). Boston Naming Test.

[bb0100] Larson J.C., Mostofsky S.H., Goldberg M.C., Cutting L.E., Denckla M.B., Mahone E.M. (2007). Effects of gender and age on motor exam in typically developing children. Dev. Neuropsychol..

[bb0105] Lee J.M., Ramos E.M., Lee J.H., Gillis T., Mysore J.S., Hayden M.R., Warby S.C., Morrison P., Nance M., Ross C.A., Margolis R.L., Squitieri F., Orobello S., di Donato S., Gomez-Tortosa E., Ayuso C., Suchowersky O., Trent R.J., Mccusker E., Novelletto A., Frontali M., Jones R., Ashizawa T., Frank S., Saint-Hilaire M.H., Hersch S.M., Rosas H.D., Lucente D., Harrison M.B., Zanko A., Abramson R.K., Marder K., Sequeiros J., Paulsen J.S., Group, P.-H. S. O. T. H. S, Landwehrmeyer G.B., Network R.S.O.T.E.H.S.D., Myers R.H., Group, H.-M. S, Macdonald M.E., Gusella J.F., HSG, C. S. O. T (2012). CAG repeat expansion in Huntington disease determines age at onset in a fully dominant fashion. Neurology.

[bb0110] Lee J.M., Galkina E.I., Levantovsky R.M., Fossale E., Anne Anderson M., Gillis T., Srinidhi Mysore J., Coser K.R., Shioda T., Zhang B., Furia M.D., Derry J., KOHANE I.S., Seong I.S., Wheeler V.C., Gusella J.F., Macdonald M.E. (2013). Dominant effects of the Huntington's disease HTT CAG repeat length are captured in gene-expression data sets by a continuous analysis mathematical modeling strategy. Hum. Mol. Genet..

[bb0115] Lee J.K., Ding Y., Conrad A.L., Cattaneo E., Epping E., Mathews K., Gonzalez-Alegre P., Cahill L., Magnotta V., Schlaggar B.L., Perlmutter J.S., Kim R.E., Dawson J.D., Nopoulos P. (2017). Sex-specific effects of the Huntington gene on normal neurodevelopment. J. Neurosci. Res..

[bb0120] Lindgren S., Koeppl G., Prinz E. (1987). Assessing child behavior problems in a medical setting: development of the Pediatric Behavior Scale. Advances in Behavioral Assessment of Children and Families.

[bb0125] Mehler M.F., Gokhan S. (2000). Mechanisms underlying neural cell death in neurodegenerative diseases: alterations of a developmentally-mediated cellular rheostat. Trends Neurosci..

[bb0130] Nithianantharajah J., Hannan A.J. (2007). Dynamic mutations as digital genetic modulators of brain development, function and dysfunction. BioEssays.

[bb0135] Nopoulos P.C. (2016). Huntington disease: a single-gene degenerative disorder of the striatum. Dialogues Clin. Neurosci..

[bb0140] Nopoulos P., Epping E.A., Wassink T., Schlaggar B.L., Perlmutter J. (2011). Correlation of CAG repeat length between the maternal and paternal allele of the Huntingtin gene: evidence for assortative mating. Behav. Brain Funct..

[bb0145] Plomin R., Deary I.J. (2015). Genetics and intelligence differences: five special findings. Mol. Psychiatry.

[bb0150] Plomin R., Mcclearn G.E., Mcgiffin P., Defried J. (2008). Behavioral Genetics.

[bb0155] Richman L., Lindgren S. (1988). Color Span Test manual.

[bb0160] Ross C.A., Aylward E.H., Wild E.J., Langbehn D.R., Long J.D., Warner J.H., Scahill R.I., Leavitt B.R., Stout J.C., Paulsen J.S., Reilmann R., Unschuld P.G., Wexler A., Margolis R.L., Tabrizi S.J. (2014). Huntington disease: natural history, biomarkers and prospects for therapeutics. Nat. Rev. Neurol..

[bb0165] Selya A.S., Rose J.S., Dierker L.C., Hedeker D., Mermelstein R.J. (2012). A practical guide to calculating Cohen's f(2), a measure of local effect size, from PROC MIXED. Front. Psychol..

[bb0170] Seong I.S., Ivanova E., Lee J.M., Choo Y.S., Fossale E., Anderson M., Gusella J.F., Laramie J.M., Myers R.H., Lesort M., Macdonald M.E. (2005). HD CAG repeat implicates a dominant property of huntingtin in mitochondrial energy metabolism. Hum. Mol. Genet..

[bb0175] Tartari M., Gissi C., Lo Sardo V., Zuccato C., Picardi E., Pesole G., Cattaneo E. (2008). Phylogenetic comparison of huntingtin homologues reveals the appearance of a primitive polyQ in sea urchin. Mol. Biol. Evol..

[bb0180] Trampush J.W., Yang M.L., Yu J., Knowles E., Davies G., Liewald D.C., Starr J.M., Djurovic S., Melle I., Sundet K., Christoforou A., Reinvang I., Derosse P., Lundervold A.J., Steen V.M., Espeseth T., Raikkonen K., Widen E., Palotie A., Eriksson J.G., Giegling I., Konte B., Roussos P., Giakoumaki S., Burdick K.E., Payton A., Ollier W., Horan M., Chiba-Falek O., Attix D.K., Need A.C., Cirulli E.T., Voineskos A.N., Stefanis N.C., Avramopoulos D., Hatzimanolis A., Arking D.E., Smyrnis N., Bilder R.M., Freimer N.A., Cannon T.D., London E., Poldrack R.A., Sabb F.W., Congdon E., Conley E.D., Scult M.A., Dickinson D., Straub R.E., Donohoe G., Morris D., Corvin A., Gill M., Hariri A.R., Weinberger D.R., Pendleton N., Bitsios P., Rujescu D., Lahti J., Le Hellard S., Keller M.C., Andreassen O.A., Deary I.J., Glahn D.C., Malhotra A.K., Lencz T. (2017). GWAS meta-analysis reveals novel loci and genetic correlates for general cognitive function: a report from the COGENT consortium. Mol. Psychiatry.

[bb0185] Vinkhuyzen A.A., van der Sluis S., Maes H.H., Posthuma D. (2012). Reconsidering the heritability of intelligence in adulthood: taking assortative mating and cultural transmission into account. Behav. Genet..

[bb0190] Zhang Y., Long J.D., Mills J.A., Warner J.H., Lu W., Paulsen J.S., Investigators, P.-H. & Coordinators of the Huntington Study, G (2011). Indexing disease progression at study entry with individuals at-risk for Huntington disease. Am. J. Med. Genet. B Neuropsychiatr. Genet..

